# A “Mechanistic” Explanation of the Multiple Helical Forms Adopted by Bacterial Flagellar Filaments

**DOI:** 10.1016/j.jmb.2012.12.007

**Published:** 2013-03-11

**Authors:** C.R. Calladine, B.F. Luisi, J.V. Pratap

**Affiliations:** 1 Department of Engineering, University of Cambridge, Trumpington Street, Cambridge CB2 1PZ, UK; 2 Department of Biochemistry, University of Cambridge, 80 Tennis Court Road, Cambridge CB2 1GA, UK

**Keywords:** bacterial flagellar filaments, flagellin, structural polymorphism, coiled coil, conformational bi-stability

## Abstract

The corkscrew-like flagellar filaments emerging from the surface of bacteria such as *Salmonella typhimurium* propel the cells toward nutrient and away from repellents. This kind of motility depends upon the ability of the flagellar filaments to adopt a range of distinct helical forms. A filament is typically constructed from ~ 30,000 identical flagellin molecules, which self-assemble into a tubular structure containing 11 near-longitudinal protofilaments. A “mechanical” model, in which the flagellin building block has the capacity to switch between two principal interfacial states, predicts that the filament can assemble into a “canonical” family of 12 distinct helical forms, each having unique curvature and twist: these include two “extreme” straight forms having left- and right-handed twists, respectively, and 10 intermediate helical forms. Measured shapes of the filaments correspond well with predictions of the model. This report is concerned with two unanswered questions. First, what properties of the flagellin determine which of the 12 discrete forms is preferred? Second, how does the interfacial “switch” work, at a molecular level? Our proposed solution of these problems is based mainly on a detailed examination of differences between the available electron cryo-microscopy structures of the straight L and R filaments, respectively.

## Graphical Abstract

**Figure:**
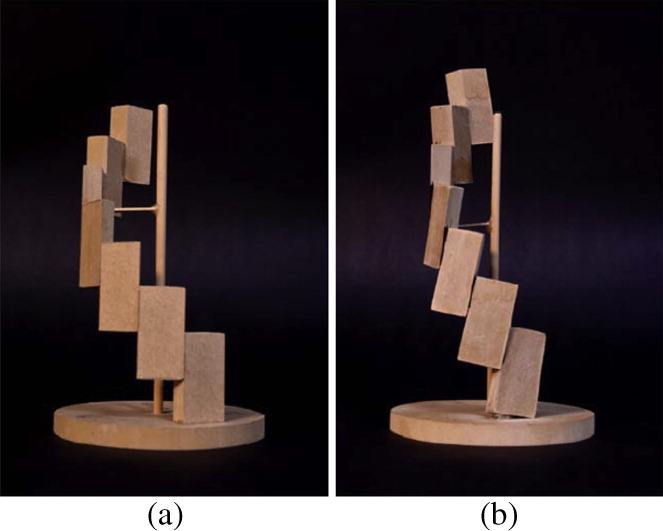

**Highlights:**

► Detailed mechanics of polymorphism in bacterial flagellar filaments. ► Selection of helical forms by amino acid substitution, mechanical torque and other factors. ► Identification of bi-stable switch at interaction between α-helices from adjacent subunits.

## Introduction

The corkscrew-shaped flagellar filaments of bacteria such as *Salmonella typhimurium*, which propel the cells through the aqueous environment when rotated by their basal motors, can adopt a range of discrete left- and right-handed helical forms.^1–5^ The filaments self-assemble from, typically, 30,000 molecules of *flagellin* protein, which organize themselves into a tube containing 11 near-longitudinal protofilaments.^6,7^

**Fig. 1:**
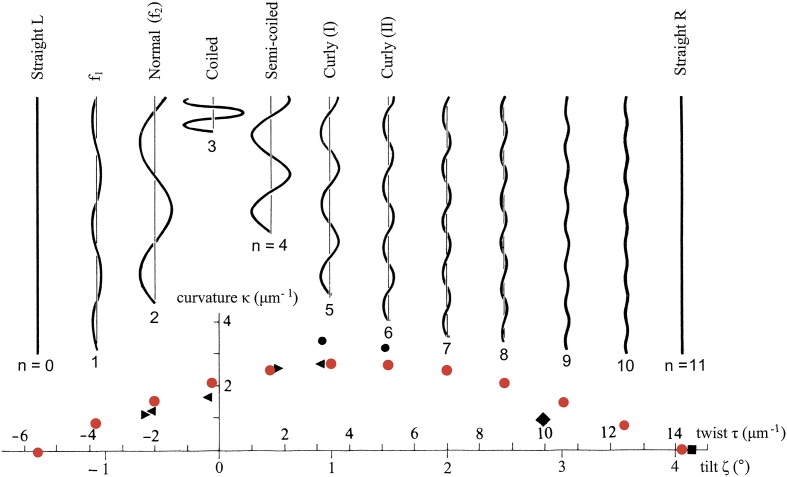
Plot of curvature κ against twist τ for measured helical forms of bacterial flagellar filaments (black points), together with the 12 discrete theoretical points (red), and drawings of the corresponding helical forms, all with contour length 4 μm (after Ref. 13). Values of twist are provided both in units of radian per micrometer and, roughly equivalently, in terms of the angle of tilt, ζ, of the 11-start protofilaments, drawn on a reference cylinder of radius 45 Å. The 12 theoretical, “canonical” states are designated by numbers *n* = 0, 1…11: *n* is the number of protofilaments in the shorter, R-type conformation. For the straight L and R filaments, *n* = 0 and *n* = 11, respectively; detailed atomic structural data at a resolution of 4 Å are available in Refs. 9 and 10.

The observed polymorphism has been explained in principle (see Fig. 1) in terms of a bi-stable molecular “switch” at the interface between neighboring molecules, in such a way that the protofilaments adopt either of two distinct states L and R, with *n* of them of type R and the remainder of type L.^1^ Extreme cases *n* = 0 and *n* = 11 are straight filaments, with distinct left- and right-handed twists, respectively; and the straight R filaments are also some 1.5% shorter than the L.^8–10^ Intermediate values of *n* provide 10 different helical forms: their twists go in uniform steps between the two extremes. Any curved rod has longitudinal “fibers”—here, protofilaments—which are shorter on the inside of the curve than on the outside; the length of these protofilaments varies sinusoidally around the circumference between the two extremes. Here, the *n* protofilaments that are switched into the shorter R form bunch together on the circumference and thus shorten that side of the filament, thereby creating curvature.^11–14^ Now, the protofilaments must also deform elastically, by small amounts, in order to satisfy the overall geometrical constraint of sinusoidal variation of length around the circumference; and indeed it is the minimization of the resulting elastic strain energy that makes the *n* shorter R protofilaments bunch together,^11–14^ thereby producing a range of just 12 different helical forms, as shown in Fig. 1. Version *n* = 2 is the “normal” left-handed helix, which is used for smooth swimming of the wild-type cells, while *n* = 5 or *n* = 6 gives “curly” right-handed helical filaments, as used in the “tumbling” maneuver of chemotaxis, when the propulsive rotary motors go into reverse.^3–5^

This entire arrangement is very different from other cooperative assemblies of macromolecular subunits, such as hemoglobin, where the switching process involves essentially identical geometrical changes at all of the equivalent interfaces between the globular subunits; that entire assembly has only two distinct states. In the tubular flagellin filaments, by contrast, a range of 12 distinct states is achieved through a bi-stable switching arrangement, by having different numbers of protofilaments switched to the shorter R conformation. Different members of the filament's family of discrete helical forms are selected by mechanical torque (such as when the filament's rotary motor goes into reverse during chemotactic maneuvers), by changes in the environmental pH, or by point mutations of the wild-type *flagellin* protein.

Although these broad principles of construction for bacterial flagellar filaments have long been understood, two specific aspects of the assembly process have remained obscure. First, precisely what characteristic of the flagellin molecule determines the value of the index *n* and, hence, selects a specific helical form for the filaments in any given case? Second, how does the switch action work in detail, so as to couple the axial shortening of the R-type protofilaments with the switch of vertical shear between adjacent protofilaments that determines a contribution to the change in twist of the filament?

This paper aims at resolving these two problems, mainly by close examination and interpretation of the detailed structures of the distinct straight L and R filaments (having *n* = 0 and *n* = 11, respectively), which have been obtained by Yonekura *et* *al*. and Maki-Yonekura *et* *al*. at 4 Å resolution, from combined X-ray crystallography and cryo-electron microscopy.^9,10^

These structural analyses of the filament identify an inner tube of mean radius 20 Å and a concentric outer tube of mean radius 45 Å. The subunits of the outer tube are larger than those of the inner tube: they consist mainly of a classical left-handed coiled coil some 80 Å long plus a shorter third α-helix at one end, whereas those of the inner tube are two α-helices some 50 Å long, making a crisscross arrangement. Kanto *et al.* have shown that single-amino-acid substitutions can dispose the flagellin protein to build helical structures with different values of *n*^15^; about half of their examples lie along the margins of the “hydrophobic stripe”,^16^ which lies at the core of the long classical left-handed coiled coil of the outer tube. Filaments can still assemble without an inner tube, but these are straight and with an over-twisted left-handed form.^17^ These and other observations suggest strongly that the answers to our two questions lie within the structure of the outer tube.

**Fig. 2:**
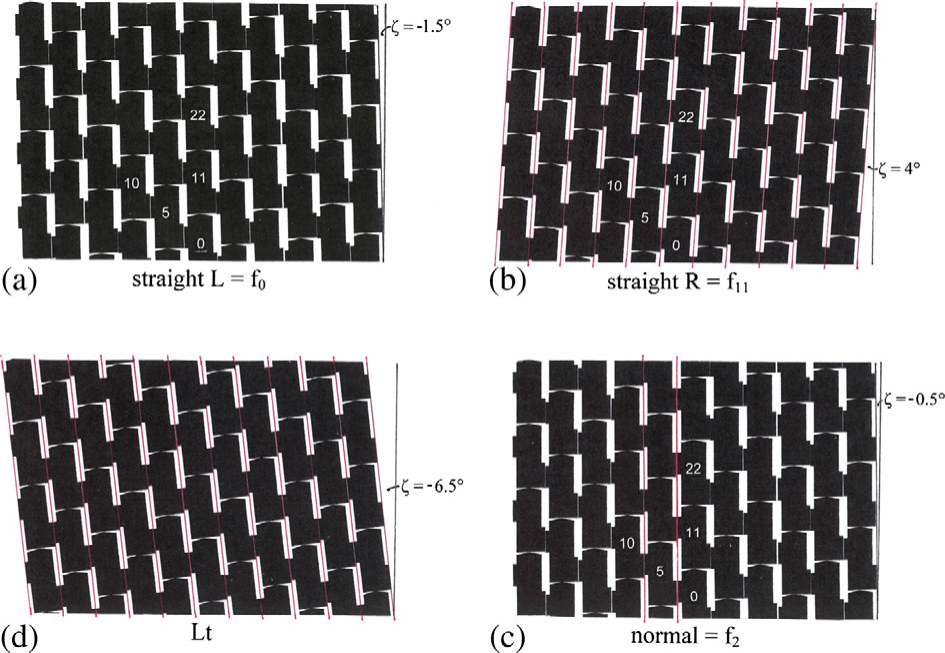
Unrolled cylindrical surface lattices of flagellar filaments at radius 45 Å. These have been constructed from schematic subunits that allow point contacts on the 11-start lines so as to make protofilaments, and extended contacts on the 5-start lines, by means of a bi-stable connection; however, there are no connections on the 6-start lines. Subunits are numbered in the order of building the filament; a selection of these numbers is shown here. Subunits 0, 11, 22 and 0, 5, 10 lie on typical 11- and 5-start lattice lines, respectively. Subunits 10, 11 and 5, 11 lie on single- and 6-start lattice lines, respectively. The 11 protofilaments may be identified as containing the subunits numbered 0, 1…10. (a) Straight filament with left-handed twist, *n* = 0: all bi-stable connections are of the L type. (b) Straight filament with right-handed twist, *n* = 11: all bi-stable connections have sheared by 2.5 Å into the R type and are marked by red lines. (c) Filament *n* = 2, the normal helical form (see Fig. 1). Here, there are two longitudinal strands of bi-stable 5-start connections of the R type—again marked by red lines—clustered together so as to minimize the overall elastic strain energy of distortion (Ref. 13). (d) Lt straight filament, formed when the polypeptide chain of flagellin is truncated so that the inner tube is not properly constructed.^8,17,18^ All bi-stable connections are of the R type; the pattern of (b) has been altered by the introduction of a shear dislocation between two adjacent protofilaments. Subunit numbers are not given here, since there is no longer a single-start lattice line passing through all subunits.

The cylindrical surface lattice of the filaments (see Fig. 2a and b) is a useful two-dimensional representation of the geometric disposition of flagellin molecules within the assembly. The lattice includes a single-start line passing through each subunit in turn, left-handed 5-start lines, right-handed 6-start lines and near-longitudinal 11-start lines or *protofilaments*. These protofilaments are tilted to the left, on a reference cylinder of radius 45 Å, by ~ 1.5° in the straight L filament, and to the right by ~ 4° in the R. Let ζ be the tilt of the protofilaments, in a clockwise sense, relative to the axis of the filament: thus ζ = − 1.5° and ζ = + 4° in L and R, respectively. This change in tilt of 5.5° in the protofilaments during the L-to-R transition corresponds approximately to an increase of 20 rad/μm in the twist of the filament (see Fig. 1.)

The layout of the remainder of the paper is as follows. First, by examining the outer-tube structures of the straight L and R filaments and differences between them, we identify a well-defined shearing bi-stable switch feature at the interface between subunits of neighboring protofilaments, with a vertical “throw” of about 2.5 Å. This feature is consistent with the fact that the twist of the filament is restricted to 12 discrete “digital” values (cf. Fig. 1). We also elucidate the nature of the bi-stability within this switch. Second, we argue that what we shall call the first-order “analog” twist of the filament—which then specifies the nearest permissible, final discrete digital value of twist—is determined by the *level of supercoiling* within the long coiled coil that is a prominent feature of the outer part of the subunit. This supercoiling can be affected, *inter alia*, by the presence of single-amino-acid substitutions close to the hydrophobic interface of the coiled coil. Third, we explain the way in which the state of the switch also controls the *length* of its neighboring protofilament, thereby endowing each of the discrete permissible values of twist of the filament with a unique curvature, according to the scheme of Fig. 1. We conclude with a general discussion. Throughout the paper, we shall explain three-dimensional geometrical ideas by means of diagrams, some of which will be highly schematic; and we shall think of the entire assembly primarily as a “mechanical system” built from bi-stable components.

## The L and R Straight Structures

Figure 2a and b shows the surface lattices of the outer tubes of the straight L and R structures, respectively, with the schematic subunits shaped so as to provide connections between them along the 11- and 5-start lattice lines but not (as is emphasized in Refs. 9 and 10) along the 6-start lattice lines. As already mentioned, in the lattices of the straight L and R filaments, the 11 protofilaments tilt, relative to the longitudinal axis, by ζ = − 1.5° and ζ = + 4°, respectively; this requires, for subunits of width 26 Å (i.e., one-eleventh of the circumference of the reference cylinder of radius 45 Å), a relative vertical sliding or shearing of about 2.5 Å. We shall take this relative displacement of adjacent subunits to be the key switch within the system that permits only the 12 digital values of twist of the canonical helical forms, as shown in Fig. 1. Figure 2c and d shows surface lattices for other forms of filament, to which we shall return later.

The simple calculation above of the nominal throw of 2.5 Å for the switch is based on a number of questionable assumptions. Thus, it ignores the shortening of the filament in the L-to-R transition; it assumes that the rotation of subunits is of the same magnitude as the rotation of protofilaments; and most seriously, it assumes that the analysis of a piece of three-dimensional machinery can be reduced to an elementary problem in plane kinematics. Nevertheless, the calculation provides an adequate approximation to the amount of relative movement at the interface, and it is suitable for other comparisons that we shall make below, mainly in relation to Fig. 2.

**Fig. 3:**
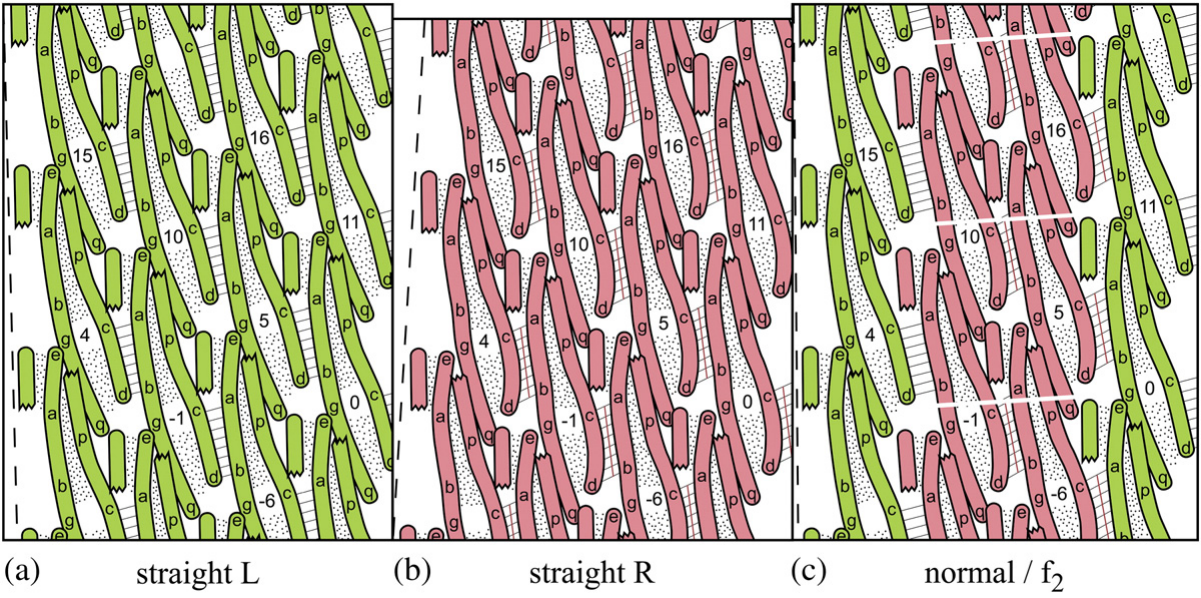
Portions of cylindrical surface lattices, as in Fig. 2, but now with subunits shown as consisting of three α-helices, representing the α-helical components of the outer-tube moiety of the flagellin subunits. The diagrams are only approximately to scale. The α-helices are shown as sausages of diameter 5 Å and are projected onto the reference cylinder of radius 45 Å. The three α-helices in a subunit are bound together as classical left-handed coiled coils by hydrophobic cores, which are here shown stippled. Within each subunit, helices ND1a (with labels e, a, b, g and q) and CD1 (with labels p, c and d) make a long classical coiled coil, while ND1b makes a short, classical coiled coil with portion ea of ND1a. The distal end of the filament is beyond the top of the picture. For the sake of clarity, the upper end of the long helix CD1 has been cut off, so as not to obscure the 11-start connection between point e at the tip of ND1a of subunit *i* and point g one-third from the bottom of ND1a of subunit *i* + 11. Likewise, the lower end of the short α-helix ND1b (not labeled) has been cut off, so as not to obscure the upper end of the bi-stable 5-start connection between the middle third of ND1a of subunit *i* (portion ab) and the bottom third of CD1 of subunit *i* + 5 (portion cd): this inter-subunit connection, which makes a right-handed coiled coil, is marked by eight short parallel lines. The broken lines on the left mark the tilt of the 11-start lattice lines. (a and b) Straight L and R filaments, respectively. The differently sheared bi-stable connections can be identified by the orientation of the short parallel lines (cf. Fig. 2). Data from Refs. 9 and 10. Note that the R straight filament is some 1.5% shorter than the L. (c) Part of the *n* = 2 filament, with two strands of bi-stable connections of type R (marked with red lines) and two protofilaments in the R form. These two protofilaments, of slightly shorter length, will only fit onto the surface of the reference cylinder—or, equivalently, onto a plane as here—if the R-type protofilaments are artificially cut and stretched, as shown. It is the pulling together of the sides of these cuts that imparts curvature to the filament.

The more detailed diagrams of Fig. 3 show the three α-helices ND1a, ND1b and CD1, which form the bulk of the outer-tube moiety of the flagellin molecule, as “sausages” of diameter 5 Å, projected radially onto the surface of the reference cylinder of radius 45 Å. We shall use the nomenclature of Refs. 9 and 10, in which the α-helical portions of flagellin's polypeptide chain occur in the order ND0, ND1a, ND1b…CD1, CD0. Here, N and C refer to the terminal regions of the chain; D0 refers to Domain 0, that is, the inner tube of mean radius 20 Å, and D1 refers to Domain 1, that is, the outer tube of mean radius 45 Å.

**Fig. 4:**
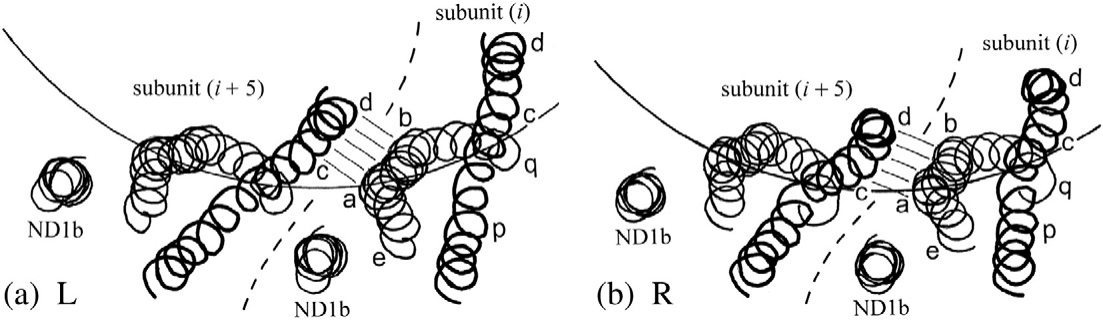
Axial view of adjacent subunits *i* and *i* + 5: adapted from a portion of Fig. 1a of Ref. 10. Only the three α-helices of the flagellin moiety that builds the outer tube are shown, as spirals passing through the α-carbon atoms. The circular arcs mark the reference cylinder of radius 45 Å. The long α-helix CD1 (cf. Fig. 3)—drawn here with a thicker line—appears more or less straight in this view, with its upper (distal) end at larger radius: locations p, c and d (cf. Fig. 3) are marked. The α-helix ND1a “wraps around” CD1 in this view: its upper end is at larger radius, and points e, a, b and q are marked. Helix ND1b, which is practically axial, appears as a small circle in this view. The bi-stable interface between ab and cd is marked by five lines, somewhat as in Fig. 3: observe that portion ab of ND1a appears to be straight in this view. (a) L filament, (b) R filament.

In the straight L and R structures at 4 Å resolution,^9,10^ the connections between subunits on the 5-start lattice lines are between the central one-third of helix ND1a and part of the short helix ND1b of subunit *i*, with parts of the lower and central one-thirds, respectively, of helix CD1 of subunit *i* + 5. In the diagrams of Fig. 3, the lower part of this connection is between portions ab and cd of the two helices and is marked by eight short parallel lines. The orientation of these short lines indicates the conformation of the switch: ab is higher with respect to cd in the red pictures, compared with the green. These 5-start connections between neighboring subunits can also be seen in the axial views of Fig. 4.

Helices ND1a of subunit *i* and CD1 of subunit *i* + 5, which constitute the lower part of the switch, form a right-handed supercoil—as distinct from the classical left-handed coiled coil mentioned earlier—and they are held together mainly by hydrogen bonds and van der Waals contacts.^9,10^ The relative axial motion within the connection is accommodated by these bonds, after rearrangement; but the bonds appear to be more numerous in the R state than in the L. The upper part of the switch, between helices ND1b of subunit *i* and CD1 of subunit *i* + 5, is also a right-handed supercoil; however, the helices are not quite so close to one another, and there appear to be relatively few potential hydrogen bonds. (Although the two maps at a resolution of 4 Å are insufficiently precise to locate every atom with certainty, they nevertheless provide a guide to the probable existence of the hydrogen bonds and van der Waals contacts.) This excess of hydrogen bonds in the R conformation of the switch suggests, somewhat awkwardly, that the contact interface on the 5-start lattice line *is more stable in the* R *than in the* L *state*.

**Fig. 5:**
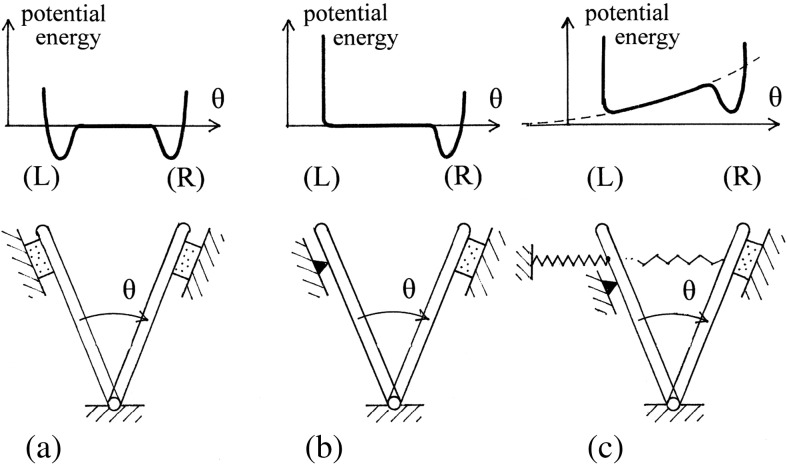
A physical analog of a mechanically bi-stable feature: the swinging gate. (a) A swinging gate with a magnetic catch (here, a stippled rectangle) at each end of its travel: the upper diagram shows a schematic plot of the total energy of the system, with two potential wells. (b) As (a), but with the magnetic catch at one end replaced by a simple stop: the arrangement is now mono-stable. (c) As (b), but with the addition of a restraining linear elastic spring that would be relaxed if the gate could swing further to the left, beyond the stop. The potential function is now the sum of two terms: a quadratic one for the elastic spring and the previous one (b) for the magnetic catch. The stiffness of the spring has here been adjusted so as to make the device bi-stable, as witnessed by the potential function.

A convenient, crude mechanical conceptual model of the kind of switch that we seek is a “swinging gate” with a “magnetic catch” at each of its two extreme positions, as shown schematically in Fig. 5a. However, in contrast, our description above corresponds to a gate with only one magnetic catch; it thus constitutes a *mono*-stable feature, as shown in Fig. 5b. Such a feature is clearly not consistent with the observed polymorphism of the filament.

This paradox may be resolved by consideration of the properties of the “Lt” structure, which Namba and colleagues have reported as being constructed when N- and C-terminal truncations of the flagellin result in an imperfectly constructed, incomplete inner tube.^8,17,18^ The Lt structure, Fig. 2d, has the protofilaments tilted at ζ ≈ − 6.5°, and it corresponds to the R structure of Fig. 2b but now altered by the insertion of a single-subunit longitudinal dislocation at the interface between two adjacent protofilaments. (Such a dislocation, of magnitude 52 Å, is equivalent to a shearing motion of 52/11 ≈ 5 Å at each inter-protofilament switch point, thus providing twice as much left-handed tilt as in the R-to-L transition.)

The Lt outer-tube structure is thus apparently more relaxed than its L counterpart; and this in turn suggests that an important role for the (complete) inner tube is to act as some sort of structural template, in order to ensure that the outer tube is “locked” into the correct surface lattice, which includes, in particular, a single-start lattice line. In terms of our swinging gate model, the L and R states are now, in effect, *prestressed* by an elastic spring (corresponding to the elastic distortion of subunits from their more relaxed Lt form), which can hold the gate in the L position, thus making the device properly bi-stable, as shown schematically in Fig. 5c, with the R state stabilized by more bonding interactions.

## Twelve Discrete Values of Twist

The bi-stable switching arrangement, as manifested in the distinct uniform L and R structures, is sufficient to explain the limited range of discrete values of twist observed between these two extremes, just as in earlier discussions of the same phenomena.^13,18^ Thus, whenever an individual longitudinal strand of 5-start connections between adjacent protofilaments is switched from type L to type R, all protofilaments increase their common tilt ζ by 0.5°; hence, when *n* of the strands of switches are in the R state, with 11 − *n* strands remaining in the L state, the tilt of the protofilaments is given by

(1) ζ=1.5°+n×0.5°,0≤n≤11

where *n* is an integer.

The bi-stability of the switches ensures that no other values of twist are permitted: it constitutes, in effect, a digital array of permitted twist values. An example of mixed L and R states of the switches is shown in Fig. 2c for filament f_2_, that is, the filament having *n* = 2 (cf. Fig. 1). This corresponds to the normal left-handed helical filament of wild-type cells, both at rest and in smooth swimming.^13^

## What Determines the Overall Level of Twist in the Filament?

So far, the only advance in the present paper over earlier mechanistic models of the filament has been to identify a bi-stable switch among the various α-helical components of the outer tube, and to provide a plausible explanation for its bi-stability.

Let us now address the question of what, precisely, determines the overall, first-order analog twist of the filament in a given case, on the presumption that the value of twist that is finally adopted by the assembly will be that permissible, discrete digital value of twist, corresponding to an integer value of *n*, which is closest to this first-order analog value. In previous studies, it has been assumed that selection of the index *n* has been due to small, unspecified overall differences in the geometry of the flagellin subunit.^11–14^ Here, we aim to be more specific.

**Fig. 6:**
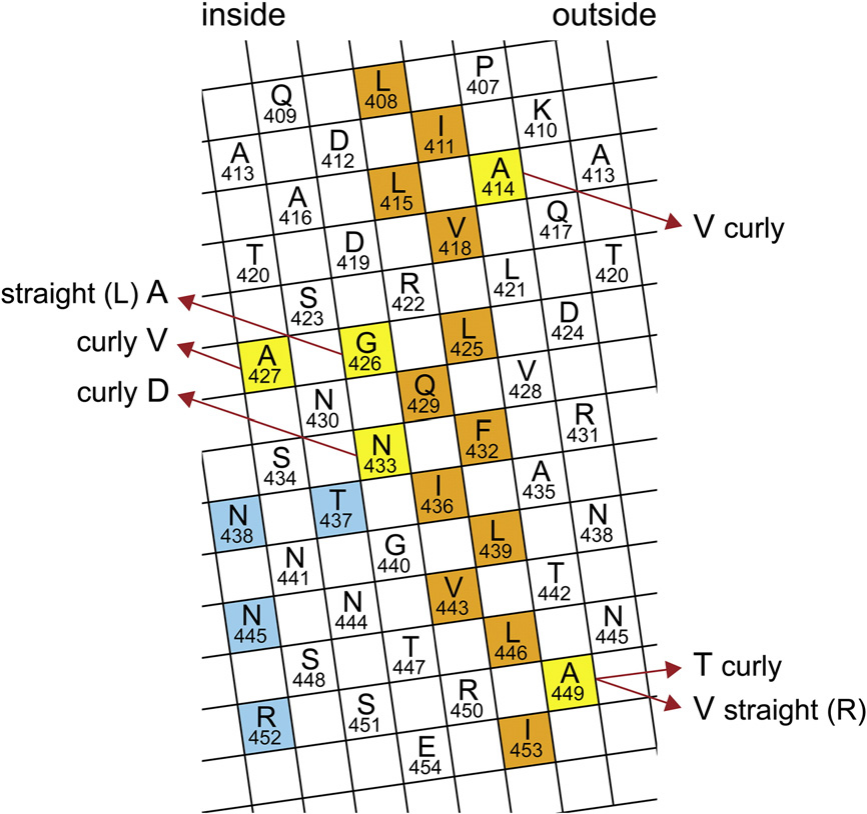
Cylindrical surface lattice of α-helix CD1 for wild-type protein, after Kanto *et al.*^15^ Here, the amino acids are represented by 5-Å squares, labeled appropriately. The diagram may be cut out and rolled into a cylinder of diameter 11 Å, ready to engage with neighboring α-helices with center–center spacing of 11 Å: note that, for this purpose, some squares are shown repeating on the edges. Residues forming the hydrophobic stripe, which connects with ND1a to make a classical left-handed supercoil, are colored orange. Six single-amino-acid substitutions that cause the flagellin to construct different helical forms have been marked.

Kanto *et al.* have presented a number of single-amino-acid mutations to wild-type flagellin, which have the effect of building different members of the discrete range of helical waveforms^15^ (see Fig. 6). We must suppose that these mutations change, slightly, the salient geometry of the flagellin building blocks, in such a way that the overall, analog twist of the filament is altered. (Recently, Hayashi *et al.*^19^ and Wang *et al.*^20^ have found many more conformation-changing single-amino-acid mutations. We shall return to a brief discussion of some of these at the end of the paper.)

In our search for understanding of such a mechanism for determination of the value of *n* in any given case, an important clue lies in the fact that, as noted above, the subunits of the outer tube contact one another along the 11-start and 5-start lattice lines *but not along the 6*-*start lines*.^9,10^

Now, an engineer who wants to build a torsionally stiff (“rigid”) tube from a number of identical subunits might well plan to have “point” contacts between neighbors on all three of the 11-, 5- and 6-start lattice lines. (Here, we think of “localized” contacts between protein molecules as being idealized as point contacts in the interests of mathematical clarity.) By a straightforward extension of Maxwell's ideas about the geometry and rigidity of structures,^14,21^ each such contact would remove 3 mechanical degrees of freedom, or 1.5 per subunit, from the assembly. A total of 6 contacts would thus remove 9 degrees of freedom per subunit: 6 as required for rigidity, and the other 3 as internal constraints on the geometry of the subunits themselves, so that the assembly will conform only to the layout of the desired surface lattice. If the point contacts on the 6-start lattice lines were now to be *disconnected*, there would remain only 4 contacts on each subunit and, hence, only 6 degrees of freedom lost—i.e., insufficient for both rigidity and the correct surface lattice.^14^ In other words, the assembly would become floppy—a “mechanism”—and thus useless as a structure. (This can readily be checked by means of simple mechanical models.) However, the situation could be redeemed, partially, by “strengthening” the 5-start connections in order to compensate for the loss of the 6-starts; that is, by replacing the 5-start point contacts by *extended connections*, of precisely the coiled-coil type that we have described above (see Figs. 2 and 3). Indeed, a feature of such a system would be that *the overall twist* of the tube could now depend upon the detailed conformation of the building blocks themselves so that a small change in the shape of the building block would produce a change in the overall twist of the assembly—a feature that is obviously relevant to the present problem.

**Fig. 7:**
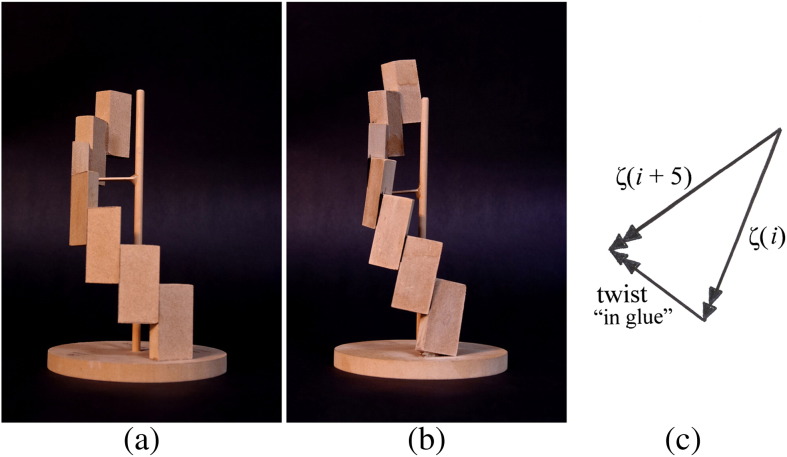
Physical models of a 5-spiral. (a) The blocklike subunits are vertical, that is, parallel with the axis of the helix. (b) The blocks are now tilted to the right by 10°, which requires the glued joints to be “twisted” by some 5°. The overall height is larger than in (a): the compensating shearing action of the “switches” between blocks has not been modeled here. (c) Vector diagram for the 10° rotation of two adjacent blocks, in a plane perpendicular to the vertical axes of (a) and (b). These vectors of “small rotation” are normal to the respective blocks, and their difference gives the “twist” of the glued joints in (b).

In order to investigate this matter further, it is instructive to consider what we might call a “5-spiral”, built by connecting building blocks together on the 5-start contacts alone, and then to regard the outer-tube structure as an assembly of five such 5-spiral structures. Figure 7a shows a set of blocks, wedge shaped in plan, and firmly glued to one another at their interfaces in order to make a rigid 5-spiral: here, all of the blocks are vertical. Figure 7b shows another assembly made from the same blocks; but now each of the blocks has been tilted to the right by ζ = 10° of rotation in its own plane, before being glued to its neighbors. In order to accomplish this, it is necessary to *twist* each glued joint by ~ 5°, as can be seen. The relationship between these two angles can be worked out from a simple vector diagram, since small rotations can be added vectorially. Thus, in Fig. 7c, 10° rotation vectors have been set out for two consecutive subunits in a plane perpendicular to the axis of the 5-spiral: they make an angle of 360°/11 = 33° with one another; and the vector difference between them (i.e., 10° × 2sin16.5° = 5.6°) corresponds to the twist or rotation required in the glued joints. In later calculations, we shall take the ratio of “twist in glue”/“change of tilt” as 0.5, for consistency with other geometric data.

This demonstration suggests that, in the case of flagellar filaments where the protofilaments rotate to the right by 5.5° (from ζ = − 1.5° to 4°) in the L-to-R transition, those geometrical changes made by the mutations that transform normal flagellin into versions that build straight L and R filaments (*n* = 0 and *n* = 11, respectively) produce alterations in geometry of the building blocks that are equivalent to an overall change of 5.5° × 5.6/10 ≈ 3° in the rotation of the glued joints in the model of Fig. 7b.

Examination of the sequence of subunits along a 5-start line in Fig. 3—e.g., subunits 0, 5, 10…—shows that, since each subunit consists primarily of a long coiled coil CD1/ND1a, the traverse from one subunit to the next along a 5-start lattice line involves the crossing of *two* superhelical interfaces: the first is within a left-handed coiled coil *inside* the subunit *i*, and the second is within a right-handed coiled coil *between* the subunits *i* and *i* + 5. Thus, we should focus attention next on any geometric changes within each of these two coiled coils during the L-to-R transition.

## Untwisting a Coiled Coil

**Fig. 8:**
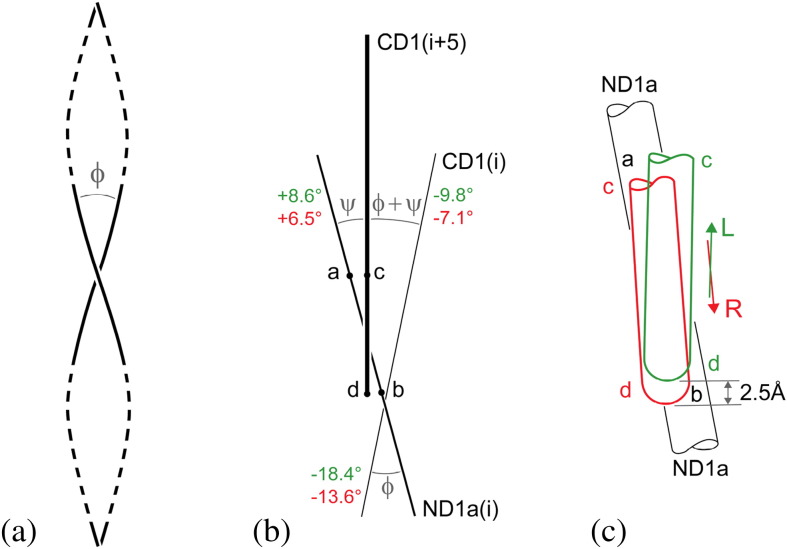
(a) Centerlines of two α-helices making a classical coiled coil and defining the crossing angle φ of the supercoil. Here, the coiled coil has left-handed twist along its axis, and by convention, φ has a negative value. Not to scale: the angle φ shown here is some 50% larger than our largest computed values. See Table 1 for the method of calculation of crossing angle. (b) Schematic view of three α-helices, looking from left to right in Fig. 3 and showing the crossing angles between them. Nearest to the viewer, and shown thickest, is α-helix CD1 of subunit *i* + 5: its lower one-third, cd, forms a right-handed coiled coil (ψ) with the central portion ab of ND1a of subunit *i*. Here [cf. (a)], the α-helices are shown straight: it is the crossing angles that are of primary interest. The crossing angle between ND1a and CD1, both of subunit *i*, is designated φ. Values of φ and ψ are given in green for the straight L structure (Ref. 10) and in red for the straight R structure (Ref. 9). Of special interest is the twist angle (φ + ψ) (“in the glue” of Fig. 7) between CD1 (*i* + 5) and CD1(*i*) and, in particular, the *change* in its value in the L-to-R transition (see Table 1). (c) Close-up view of the switch connection ab/cd of (b). The portions cd shown in green and red correspond to the straight L and R structures, respectively: note that the switch connection is such that the change in angle ψ is directly related to the vertical shearing movement of 2.5 Å within the switch. Data from Refs. 9 and 10.

In general, the degree of supercoiling of a coiled coil may be characterized by the “crossing angle” φ made by the centerlines of the two α-helices. This is shown in Fig. 8a for a regular, idealized coiled coil, and we shall regard φ as being positive in a right-handed supercoil.

Table 1 presents average values of crossing angle for these two distinct coiled coils in each of the straight L and R structures: details of the method of calculating crossing angles are given in the legend of Table 1. We designate φ and ψ as the crossing angles within and between the subunits, respectively, as shown schematically in the view of Fig. 8b. Here, each α-helix is represented by a straight line; such a simplification does not invalidate our calculation of overall *changes* in angle during the L-to-R transition. The final result, that is, the sum of the changes in φ and ψ during the L-to-R transition, is 2.7°, which is in fair agreement with the estimated value of 3° obtained above. All of this suggests, therefore, that what primarily drives the overall, first-order analog change of twist of the filament during the L-to-R transition is the *change of supercoil* in the long CD1/ND1a coiled coil, which in turn is a consequence of the single-amino-acid substitutions mentioned already.

Now, there is also a significant change of crossing angle within the shorter, right-handed coiled coil that constitutes the 5-start switch connection between subunits *i* and *i* + 5. We shall argue below that this change is directly related to the action of the switch, as described above; and that it is specifically the additive combination of these changes in both φ and ψ that determines the overall, first-order analog twist of the filament.

Figure 6 shows an “unrolled” map of α-helix CD1 for wild-type flagellin, with its prominent classical hydrophobic stripe^16^ colored orange. As remarked by Kanto *et al.*, in general, those mutations that change the helical form of filaments from that of the wild type lie just outside this hydrophobic stripe.^15^ Thus, mutations G426A and A449V, which produce L and R filaments, respectively, obey this rule—as indeed do A449T, A414V and N433D, which all produce curly filaments. The mutations of this group that involve substitution of one hydrophobic amino acid by another also satisfy a second rule: that a change to a *larger* amino acid on the “outside” margin—i.e., the margin lying further from the axis of the tube—*increases* the twist of the filament, whereas a larger hydrophobic amino acid on the “inside” margin *decreases* the twist. [We offer no comment here on the prospective generality of such a rule or on the mutations N433D (an exchange of *polar* residues) and A427V (which is not adjacent to the hydrophobic stripe).]

It ought not to be surprising that such changes can affect the overall superhelical twist of a “classical” coiled coil, with its hydrophobic core. For example, in the case of the TolC tube assembled from 12 α-helices, it is known that changes in size of the hydrophobic amino acids lying *within* the hydrophobic stripe make even larger changes to the crossing angles of the coiled coils—about double those experienced here.^22^

In summary, we suggest that variations in the twist of flagellar filaments—as finally “made digital” by the inter-subunit switch system into one of the twelve permissible values—are a direct consequence of changes in supercoil of the long CD1/ND1a coiled coil, as occasioned by mutations. Later on we shall discuss other shape-changing mutants described by Kanto *et al.* that do not feature in Fig. 6,^15^ and also some recent results obtained by Hayashi *et al.*^19^ and Wang *et al.*^20^ We shall also discuss several other well-known ways in which changes in the helical waveform of flagellar filaments may be produced—e.g., by changes in pH of the environment or mechanical torque.

## What Determines the Curvature of a Flagellar Filament?

It has long been accepted, in principle, that the curvature of flagellar filaments is a direct consequence of a switching system of the type described above. Thus, it is recognized that any of the 11 protofilaments that switch from the L state to the R state also experience an intrinsic shortening by some 1.5%; and also that it is this pattern of shortening of the R-type protofilaments—bunched together so as to minimize the overall elastic strain energy of the assembly^12^—that produces the well-known half-wave canonical pattern of discrete states of curvature and twist shown in Fig. 1.

What, then, is the machinery for *shortening* protofilaments of the R type when a switch occurs? In seeking to answer this question, we can obviously examine the details of the straight L and R filaments for clues; but crucially, we shall also need to develop from the known uniform straight L and R structures an understanding of how individual R-type filaments, *even when they are adjacent to L*-*type protofilaments*, also become shorter. This is our final major task.

Now, Namba and colleagues have pointed out that the 11-start connection between the upper tip of α-helix ND1a of subunit *i* and a point one-third from the bottom of ND1a of subunit *i* + 11—points e and g in Fig. 3—undergo a relative horizontal movement of a few angstrom units in the L-to-R transition (Fig. 4e of Ref. 10), in addition to the relative vertical movement of 0.8 Å that is responsible for shortening the R filaments as a whole.

Examination of the structures of Refs. 9 and 10 shows that there are indeed two other local contacts between successive flagellin molecules along the 11-start protofilaments. The first of these is physically close to the contact described above: it is between a point one-third down CD1 of subunit *i* and the lower tip of ND1a of subunit *i* + 11—shown by points p and q in Fig. 3. Here again, the vertical relative movement of 0.8 Å is accompanied by a horizontal relative movement of a few angstrom units. The third local contact between subunits *i* and *i* + 11 is between the lower tip of CD1 of subunit *i*—point d in Fig. 3—and the lower tip of α-helix ND0 of subunit *i* + 11, which lies on the inner tube of the filament and is not shown in Fig. 3 (but see Fig. 5 of Ref. 9 for a stereoscopic view of the contact region). Here, we shall focus attention on the connections e/g and p/q.

**Fig. 9:**
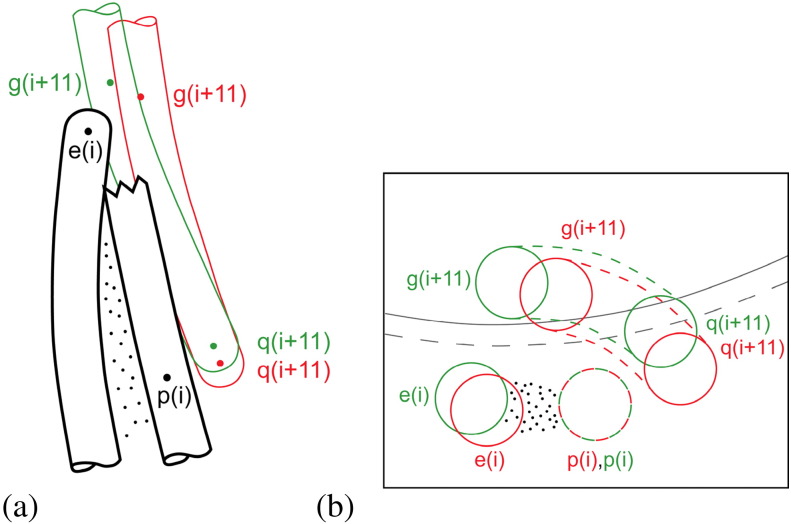
Schematic local close-up views of the 11-start interconnections between portions e and p of subunit *i* and portions g and q of subunit *i* + 11. In (a), close-ups of a single connection in the straight L and R conformations (from the same viewpoint as for Fig. 3) have been superposed so that the relative motion of subunit *i* + 11 to subunit *i* may be seen. In this picture, the lower portion of α-helix CD1 of subunit *i* + 11 has been shown green (L) and red (R); the relative vertical movement (which makes the R protofilament significantly shorter than the L) has been exaggerated by a factor of 2, for clarity. In (b), an axial view (at a larger scale: 1.5 ×) of the same feature: the circular arcs are at radius 45 Å, and the pictures have been superposed on portion p. Here, we can see that the relative movement p/q is mainly radial, while at e/g the relative movement is mainly circumferential. Data from Refs. 9 and 10.

These two movements are detailed in the schematic views shown in Fig. 9. Figure 9a shows enlarged portions of Fig. 3a and b but now superposed so as to show the movement of g and q relative to e and p in the L-to-R transition: in the axial view of Fig. 9b, the contacting parts of the α-helices are represented by circles. It seems clear that the two relative movements described above are coupled, since points g and q on subunit *i* + 11 are not far from one another along the same α-helix; while points e and p are on helices that combine to make a classical coiled coil. Observe, in particular, in Fig. 9b that the horizontal component of the relative movement of p/q is mainly *radial*, while that of e/g is mainly circumferential; and that q(*i* + 11) (i.e., q of subunit *i* + 11) moves radially *outwards* relative to p(*i*)—as indeed must q(*i*) relative to p(*i* − 11)—in the L-to-R transition. In turn, this means that q(*i*) moves radially outwards relative to p(*i*) or, in other words, *each subunit rotates by a small angle about a horizontal axis tangential to the reference cylinder* in the L-to-R transition. The relative radial motion of p and q is roughly 3 Å, and these two points on a given subunit are separated axially by about 50 Å; hence, the angle of rotation is about 3°, which is in rough agreement with the figure of 2.1° in Table 1.

We claim that such a rotation is consistent with, and *indeed is directly due to*, the change in crossing angle ψ at the 5-start connection between subunits, in the L-to-R transition (see Fig. 8c). Here, then, is a mechanism for making a protofilament *i* + 5 *shorter* than its neighbor *i* when the interface between them is of type R. This is shown schematically in Fig. 3c, where, as in Fig. 2c, the interface between the subunits of protofilaments 0 and 5, as well as between those of protofilaments 5 and 10, is of type R, while the other 5-start interfaces are all of type L. The relative vertical movement of the switches in the R conformation—marked with red lines—rotates cd relative to ab, in the sense indicated in Fig. 8c. This pushes point p inwards relative to point q in the subunits colored red in Fig. 3c; which in turn lifts e (axially) relative to g in these subunits by means of a coupled horizontal and vertical relative motion within the e·p/g·q connection. The details of this feature are not yet fully understood; however, in our view, the arrangement is likely to be “passive”, without involving any bi-stability. This action shortens the two red-colored protofilaments and makes the filament itself curved. In this picture, we have introduced an artificial *cut* across the red (R-type) subunits so that the whole assembly can be unrolled onto a plane: it is the closing-up of these gaps that will “pull” the filament into its actual curved shape, thereby adjusting elastically the axial lengths of all protofilaments so that they vary as cosine 2θ around the circumference of the filament, as required by Kirchhoff's hypothesis (see Ref. 12, Appendix).

## Why Does the Sliding of the Switch Also Twist It?

We have already discussed the bi-stable up-and-down shearing motion of the extended 5-start connection between subunits; thus, we should now explain why there is a *coupled* reduction in its right-handed superhelical twist when the switch moves from type L to type R, as shown in Fig. 8c. We propose here a mechanism that is suggested by a close examination of the a(*i*)/c(*i* + 5) and b(*i*)/d(*i* + 5) local connections that can be seen in the data of Refs. 9 and 10, and an inspection of putative hydrogen bond links between the components. An observation in relation to the a/c connection is that residue E83 of a would appear to make hydrogen bonds with each of S434 and N438 of c *in both* L *and* R *positions*. If that were indeed the case, the inclination of line S434–N438 to the axis of α-helix CD1 would couple an up/down relative motion of the switch to a radial in/out relative motion of the sense given in Fig. 8c. In the apparent absence of such a linking feature in the b/d connection (Fig. 8b), we tentatively suggest that this special a/c linkage could be responsible for the coupled sliding/twisting action within the coiled-coil switch, as observed.

## Interaction of Superhelical Twists φ and ψ

In our examination of differences between the L and R straight structures, we have found, in particular, changes in the superhelical twist of two different coiled coils: φ in the long left-handed ND1a/CD1 coiled coil within the subunit and ψ in the shorter ND1a(*i*)/CD1(*i* + 5) right-handed coiled coil at the interface between adjacent subunits on the 5-start lattice lines (see Table 1). Also, we have explained the change in protofilament tilt ζ between the L and R structures in terms of the change in the sum of φ and ψ.

We now need to explain how, in general, a change in φ, in consequence of a point mutation or some other cause, can produce a distinct change in value of the parameter *n*, in order to determine both twist and curvature of the filament. This will require a little algebra.

We start with data from Table 1 and write

(2) φ=−18.4°+γ×4.8°

(3) $$\psi =8.6{}^{\circ}\;-\;\left(n/11\right)\times 2.1{}^{\circ}$$

In Eq. (2), we have introduced a dimensionless measure, γ, of the change in φ relative to its value in the straight L structure so that values γ = 0 and γ = 1 correspond to the L and R structures, respectively. Our basic problem now is to find a relationship between γ and the helix-selection index *n*.

Equation (3) expresses the fact that individual values of ψ can only be either 8.6° or 6.5°, depending on whether the corresponding switch is in the L or R state, respectively. However, note that Eq. (3) gives the *average* value of ψ for all 11 protofilament interfaces. We need the average here because, although the value of φ + ψ depends on whether the switches are in the R or L state, the tilt of all protomers must be equal. This calls for some elastic re-adjustments within the subunits, and use of the average value of ψ here.

We now write down two independent expressions for the tilt angle ζ. The first is a repeat of our earlier formula for ζ in terms of the number *n* of protofilament interfaces that have been switched into the R state:

(1, bis) ζ=−1.5°+n×0.5°

The second expression relates the tilt ζ to the *change* in the sum of φ and ψ [by use of Eqs. (2) and (3)] for given γ and *n*, in accordance with the geometry shown in Fig. 7:

(4) $$\zeta =-1.5{}^{\circ}+2.0\left(4.8{}^{\circ}\times \gamma \;-\;\left(n/11\right)\times 2.1{}^{\circ}\right)$$

Here, we have used a value of 0.5 for the ratio of the change in “twist in the glue” to change in ζ, as mentioned above, thus giving the multiplier 2.0 in Eq. (4). This has been done so that ζ changes by + 5.4° in Eq. (4), when going from L (γ = 0, *n* = 0) to R (γ = 1, *n* = 11), in order to be close to our working value of 5.5°.

Eliminating ζ between Eqs. (1) and (4) and rearranging, we obtain, finally,

(5) $$\gamma =\left(n/10.9\right)\approx \left(n/11\right)$$

In other words, changes in *n* are directly proportional to changes in the crossing angle, φ, in the long ND1a/CD1 coiled coil. The “approximate equality” sign (≈) here covers both a negligible difference between 11 and the precise constant in Eq. (5); and also the fact that *n* must be a positive integer while γ, like φ, is in principle a continuous variable. The chosen value of *n* is thus the integer closest to 11 γ.

The result in Eq. (5) may seem to be intuitively obvious, but the above calculation is necessary not only because φ can, in principle, change continuously, but also because the analysis reveals that we need to use the average value of ψ around the filament's circumference—which presupposes some general elastic rearrangement within the assembly.

## Discussion

In this paper we have assembled what we consider to be the salient features from a range of experimental sources, in order to construct a model that provides answers to the questions: (a) what determines the value of *n* and (b) how does the switch work?

We have appealed to numerical values of key variables in various places; but, we emphasize that our explanations are essentially qualitative, in the sense that they do not hang critically on precise numerical values.

Our key idea is that the *tilt* of subunits and of the protofilaments, and hence the analog twist of the filament, is determined primarily by what appears as “the twist in the glue” in the physical model of the 5-spiral structure shown in Fig. 7. This twist in the glue is the sum of changes in the crossing angles of two different coiled coils, which behave independently. The crossing angle φ within the long classical supercoil CD1/ND1a of the outer part of the subunit is influenced by the presence of mutations close to the hydrophobic stripe—and indeed by other factors that we shall consider below. In contrast, the crossing angle ψ within the 5-start switch connection between adjacent subunits depends only upon which of the two possible stable positions is adopted by the switch. The overall tilt ζ of protofilaments determines how many, *n*, of the 11 strands of switches are to be in the R state, according to the geometry that is epitomized in Eq. (1). The change in crossing angle of the switch also activates the shortening of the R protofilaments, which in turn determines the overall curvature and hence the discrete helical form of the filament.

The entire tubular structure is a subtle assembly of interconnected elastic subunits. The bi-stability of the switch depends in part on elastic prestress of the subunits that, in the absence of the constraint of the inner tube, are more relaxed in the different Lt construction, with its higher left-handed twist, by reason of the long classical coiled coil's adopting a more relaxed form, with a larger left-handed crossing angle.

In concentrating on the role of the switch, we may have tended to over-idealize the various structural components of the system as being more or less rigid. However, we know that the geometry of curved rods requires lengths of the protofilaments to vary sinusoidally around the circumference, as mentioned in Introduction above, according to Kirchhoff's hypothesis, when the filament is curved. Indeed, we must invoke a principle of “minimum elastic strain energy” in order to produce the “clustering” of R filaments that is necessary to provide the overall empirical relationship between curvature and twist, as displayed in Fig. 1.^11–14^ At present, it does not seem feasible to be more precise than this about the role of elasticity within the assembly and its components.

Now, a significant minority of the form-changing single-residue mutations in the study of Kanto *et al.* were located on the *inner tube* of the structure.^15^ Since, as remarked above, it does not seem feasible to fit any “switching” machinery into the inner tube, we suggest that the role of inner-tube mutations may be to alter the *intrinsic twist* of that tube and, in turn, to exert a “twisting prestress” onto the outer tube. In this connection, we observe that the inner-tube mutations in Ref. 15 change the value of index *n* by no more than 1, from 2 to 3. Such an outcome is consistent with the consideration that the torsional stiffness of the inner tube is likely to be an order of magnitude smaller than that of the outer tube. Note, however, that Hayashi *et* *al*. find that some inner-tube mutations have stronger effects than these (Hayashi *et al.*, unpublished results).

A notable feature of the study of Ref. 15 is that 5 of the 15 mutations described there change the normal filament into *straight* filaments with left-handed twist. It seems quite possible that these different individual mutations change the left-handed analog supercoil of the long coiled coil by different amounts, which are all at least as large as that required to provide the L straight filament. (Three of these mutations lie on α-helix ND1b, which we have not considered in detail.) In other words, an *over*-*large* change in left-handed supercoil cannot construct a filament *beyond* the straight L filament along the negative twist axis: the digital value *n* = 0 may correspond in principle to a range—possibly wide—of analog left-handed twist. The same consideration may well apply at the other end of the spectrum, since no helical form can exist beyond the straight R kind, *n* = 11.

These considerations may help to explain how it is that mutations A449V and G426A, which separately produce straight R (Δ*n* = + 9) and straight L (Δ*n* = − 2) filaments when applied respectively to wild-type (SJW 1103) flagellin, produce curly filaments (Δ*n* = 3 or 4) when they are applied simultaneously.^19^ Here, we define Δ*n* as the change in value of index *n* relative to its value (*n* = 2) for the wild-type filaments. In other words, the absence here of a simple addition of effects—which might be inferred from our description of the workings of the molecular machinery as a whole: it would suggest Δ*n* = + 7 here—could be due to such presently unknowable “end effects”.

Another observation of Kanto *et al.* is that, while mutation A449V applied to wild-type flagellin produces straight R-type filaments, as noted above, mutation A449T produces curly filaments; thus, Δ*n* = 9 and Δ*n* = 5 or 6, respectively, in the two cases (see Table 2).^15^ Here, then, changes from a hydrophobic amino acid to another hydrophobic, and to an uncharged polar one, produce significantly different changes in value of the index *n*. These data are compatible with our central idea that alteration of the characteristics of individual amino acids can affect the superhelical shape of coiled coils.

Hayashi *et* *al*.^19^ have recently studied the effects of many more mutations than Kanto *et al.*^15^ by starting with non-motile mutants—having either straight or curly filaments—in a dish of nutrients. Over a period of 2 days, mutations into motile forms occur spontaneously. Examination of the DNA of such mutants, and of the changes in flagellar waveform that they produce, have provided information on the order of 10 mutation sites on each of the α-helices ND1a, ND1b and CD1 and on the order of 5 sites on each of ND0 and CD0, in addition to several sites on the near-radial “spoke” connecting the upper end of ND0, of the inner tube, to the lower end of ND1a, in the outer tube, and other locations not on α-helical coiled coils. It may be possible in future to draw some positive conclusions by study of this wealth of data. For the present, however, we report a few striking instances from Hayashi's work of somewhat surprising phenomena, whose explanation would require levels of structural detail beyond those that have been available in the present study.

Several mutations where the substitution of an amino acid by one of the same general kind (i.e., hydrophobic to hydrophobic or hydrophilic to hydrophilic) have been found to produce significantly different changes, Δ*n*, from the substitution of the same amino acid to one of the opposite kind—as in the example of A449V and A449T, cited above.

Also, mutations A427V, A427T and A427G, when applied to flagellin SJW 1660 (i.e., SJW 1103 + mutation G426A, with straight L filaments), all restore filaments to the normal kind; but the normal filaments produced by A427T in particular do not transform under either mechanical torque or changes in pH or salt concentration in the environment. Hence, this particular mutation appears to raise significantly the potential energy barrier within the switch mechanism, in a way that is currently hard to understand.

Again in relation to the straight filaments of SJW 1660, mutations R431C and R431H produce normal filaments, but R431S produces large-radius “non-canonical” filaments, with twist similar to that of semi-coiled (Fig. 1) but with curvature ~ 1 μm^− 1^, that is, only half of the canonical value. Wang *et* *al*. have also reported “non-standard” filaments that have approximately double or half of the curvature of canonical forms having similar twist values.^20^ Notably, some of their mutations are near to the localized connections between subunits *i* and *i* + 11, which could conceivably affect the length changes as between L- and R-type protofilaments and, hence, the resulting curvature of the filaments. However, it is difficult to see that such an explanation could apply to the mutation R431S, mentioned above.

These recent, varied observations pose a challenge for future studies of flagellar polymorphism. Explanations of such puzzling phenomena would need to go beyond the ideas expressed in the present paper.

Our detailed postulated explanation of the mechanics of polymorphic switching in flagellar filaments suggests some experimental tests by means of artificial substitution of specific amino acids of flagellin. One of these involves the replacement of glutamic acid 83 by alanine (i.e., mutation E83A). This would prohibit the formation of the hydrogen-bonded triangle that we have postulated above to be responsible for coupling a change of twist with the 2.5-Å vertical switch at the interfaces on the 5-start lattice lines—with serious consequences for the overall mechanics of polymorphism.

This brings us to the well-known fact that there are several different ways, in addition to the provision of point mutations, of making flagellar filaments change their helical shape to that of another member of the family of discrete helical forms. These include:

(a) changes of pH and ionic strength of the environment,^23–26^ changes in temperature^27^ and the presence of alcohol^28^;
(b) co-polymerization of monomer from different strains of bacteria^1,29,30^ and
(c) the application of mechanical torque^4,5,31,32^ or the application of electric fields.^33^

That changes in pH can alter the value of *n* is not surprising: they tend to change the strength of ionic interactions of titratable groups and hence, in principle, the “tightness” of classical coiled coils. The other factors mentioned in (a) may well have a similar effect.

An example of scheme (b) is the co-polymerization of two monomers that build, separately, straight L and R filaments.^30^ Mixing of these monomers in different proportions produces canonical helical filaments with a range of different values of the index *n*. Here, one possibility is that the two types of monomer are distributed uniformly over the surface lattice. Another is that each separate protofilament is built from a single type of monomer. In either hypothetical scenario, it is not difficult to understand in principle how the mixing of monomers could lead to different helical flagellar forms.

Perhaps, (c) is the most straightforward situation to understand. Provision of an external torque—as when the rotary motors go into reverse during the tumbling phase of chemotaxis^4,5^—will oblige the elastic outer tube to adopt a different analog value of twist; this will require a different number, *n*, of protofilaments to switch from the L to the R form, with consequent changes in curvature. Similarly, the application of an electric field^33^ will align dipoles and thereby exert a distorting effect on the filament.

None of these phenomena poses a new problem in understanding the mechanics of the switching system.

Switching actions are, of course, widespread in biological molecules.^34^ The distinctive feature of the present application in bacterial flagellar filaments is the capacity of the mechanical system *to adopt several different helical forms*, having distinct propulsive characteristics when rotated by their motors in the aqueous environment.

In our earlier work, which made a specific proposal for a workable conceptual model for bacterial flagellar filaments—but which also required the diameter of the filament to change significantly with *n*, contrary to structural data that have emerged subsequently—attention was paid to the clear requirement that alterations in the state of the inter-element switches *must propagate only along the 11*-*start lattice lines*.^13^ That particular study was made on an assembly of elastic elements forming a *plane*, unrolled version of the tube. At that time, it was not appreciated that it is much more straightforward to ensure propagation along 11-start lines—in the present work by sympathetic shearing of lines of switches in the 11-start direction, as in Fig. 2—in a *cylindrical*, as distinct from a *plane* geometry.

Finally, we observe that, in our proposed explanation of the construction of polymorphic bacterial flagellar filaments, *helical structural motifs* are used at four different constructional levels, as follows: first, the α-helical folding of the polypeptide chain; second, the coiled-coil “superhelical” assemblies of pairs of α-helices; third, the 5-spiral structures; and fourth, the helical forms of the filaments themselves. Such a scheme for building a hierarchy of spiral structures is a *tour de force* of molecular evolution.

## Figures and Tables

**Table 1:** Crossing angles of coiled coils (cf. Fig. 8)

Coiled coil	φ [CD1(*i*)/ND1a(*i*)] (°)	ψ [ND1a(*i*)/CD1(*i* + 5)] (°)
Straight L	− 18.4	+ 8.6
Straight R	− 13.6	+ 6.5
Change: L to R	+ 4.8	− 2.1
Overall inter-subunit change = + 4.8 − 2.1 = + 2.7		

These crossing angles were computed as follows, from data in Refs. 9 and 10. First, approximate coordinates of points on the centerlines of the α-helices were computed at five-residue steps along the helices from coordinates of the successive C^α^ atoms, by use of a simple weighted-mean formula. The crossing angle between straight lines joining two of these centerline points from each of the two α-helices was obtained by first finding the line segment perpendicular to both of these lines and then computing the angle between the lines, as viewed along this segment. The value of the crossing angle produced in this way depends to some extent on the specific centerline points chosen from the set of such points at five-residue spacing along each of the helices—particularly, if the coiled coil is irregular. The values given here were found from centerline points near the ends of the respective coiled coils. The longer, ND1a/CD1, coiled coil corresponds broadly to the portion shown in the schematic diagram of Fig. 8a by continuous lines; it is clear that this method would underestimate the value of φ by the order of 10% for a perfectly uniform coiled coil. However, these coiled coils are not uniform; the calculation of *change* in “end-to-end” crossing angle between the L and R structures seems likely to be reasonably reliable.

**Table 2:** Mutations of wild-type flagellin (SJW 1103), applied either singly or as a pair: Δ*n* − 2 is the change in value of *n*, relative to the wild type (*n* = 2)

Strain	Mutation	Helical form	*n*	Δ*n* − 2
SJW 1655	A449V	Straight R	11	9
SJW 1660	G426A	Straight L	0	− 2
SJW 1660-D 503	A449V + G426A	Curly	5	3
SJW 2871	A449T	Curly	5 or 6	3 or 4

Data in rows 1, 2 and 4 are from Ref. 15, and those in row 3 are from Ref. 19.
